# Commentary on Ferguson, et al., “Impact of Non-pharmaceutical Interventions (NPIs) to Reduce COVID-19 Mortality and Healthcare Demand”

**DOI:** 10.1007/s11538-020-00726-x

**Published:** 2020-10-07

**Authors:** S. Eubank, I. Eckstrand, B. Lewis, S. Venkatramanan, M. Marathe, C. L. Barrett

**Affiliations:** 1 Biocomplexity Institute and Initiative, University of Virginia, Charlottesville, USA; 2 Department of Public Health Sciences, University of Virginia, Charlottesville, USA; 3 Department of Computer Science, University of Virginia, Charlottesville, USA

**Keywords:** Epidemiology, COVID-19, Non-pharmaceutical intervention, Compartmental model

## Abstract

A recent manuscript (Ferguson et al. in Impact of non-pharmaceutical interventions (NPIs) to reduce COVID-19 mortality and healthcare demand, Imperial College COVID-19 Response Team, London, 2020. https://www.imperial.ac.uk/media/imperial-college/medicine/sph/ide/gida-fellowships/Imperial-College-COVID19-NPI-modelling-16-03-2020.pdf) from Imperial College modelers examining ways to mitigate and control the spread of COVID-19 has attracted much attention. In this paper, we will discuss a coarse taxonomy of models and explore the context and significance of the Imperial College and other models in contributing to the analysis of COVID-19.

Infectious disease epidemiologists’ workhorse mathematical model throughout the twentieth century was the compartmental model, which partitions a population into a small set of possible disease states, e.g., susceptible (*S*), infectious (*I*), and removed (*R*), and specifies transition rates among the states. Typical models have three general properties:

- The rate of transition from a susceptible state, *S*, to an infectious state, *I*, is proportional to the product of the number or fraction of people in each, $\beta \cdot S \cdot I$.
- The rate of transition out of the infectious state, $\gamma \cdot I$, sets a timescale for the model.
- The overall growth in infections is proportional to the ratio of these transition rates.

Compartmental models reproduce observed features of outbreaks, such as a self-limiting period of nearly exponential growth to a single peak followed by gradual decrease as the pool of susceptibles (*S*) in the population is depleted.

The simplest compartmental models have only a few parameters, and general characteristics of an outbreak are amenable to straightforward mathematical analysis. For example, consider the so-called *S*–*I*–*R* model:

$$ {\text{d}}S/{\text{d}}t = - \beta SI;\quad {\text{d}}I/{\text{d}}t = \beta SI - \gamma I;\quad {\text{d}}R/{\text{d}}t = \gamma I. $$

Because the product $S \cdot I$ vanishes when *either S* or *I* vanishes, these are fixed points of the dynamics. That is, when there are no susceptible or infectious people, the outbreak is over. However, the fixed point at *S *= 0 is stable, while the one at *I *= 0 is unstable. For a disease in which infected people may become immune, a perturbation out of the state with nobody infected (*I *= 0) leads eventually to the state with no susceptibles remaining (*S *= 0). Deterministic compartmental models have stochastic cousins such as the Reed–Frost model (Abbey 1952), which allow for the possibility of stochastic outbreak extinction. In stochastic models, the outbreak may reach the *I* = 0 fixed point in finite time without completely exhausting susceptibles (*S* > 0).

Infectious disease outbreaks are essentially chain reactions in which each infectious person turns susceptibles into infecteds. The rate at which new infections occur is determined by a dimensionless combination of model parameters known as the *reproductive number*, ***R***. The reproductive number can be interpreted as the mean number of people who will be directly infected by a typical infectious person over the course of illness. When $\varvec{R} > 1$, disease prevalence increases exponentially. For example, in the *S*–*I*–*R* model, the equation for *I* can be rewritten as

$$ {\text{d}}\ln  I /{\text{d}}t = \beta S - \gamma . $$

At any instant, *I* is increasing or decreasing exponentially fast, depending on whether $\varvec{R} = \beta S/\gamma$ is greater or less than one. Typically, as in this simple model, there are three ways to reduce ***R***:

1. Reduce *S*, the number of susceptibles, usually through prophylaxis;
2. Reduce *γ*^−1^, the duration of the infectious period, through treatment;
3. Reduce *β*, the rate of transmission, through physical or behavioral barriers.

The *basic* reproductive number, $\varvec{R}_{0}$, is the reproductive number in a situation in which the entire population is susceptible and no mitigating interventions are in place. Although the basic reproductive number is often treated as a biologically determined characteristic of the pathogen’s transmissibility, it is actually a combination of biological, environmental, behavioral, and social characteristics, including relative transmissibility via different pathways (e.g., transmission by airborne droplets vs. transmission via surfaces or body fluids); local weather’s effects on the ability of the virus to survive and/or infect new hosts; and the host’s contacts with other potential hosts. Usually, only a few of these factors are included explicitly in compartmental models, limiting their ability to represent mitigating interventions. In particular, social distancing aims to reduce *β* by limiting the number of contacts between infectious and susceptible people in which transmission can occur.

It is important to distinguish between “flattening” the epidemic curve, i.e., reducing *I* at all times, and “moving the curve to the right,” i.e., delaying the peak of *I*. The impact of the epidemic can be substantially mitigated if the demand for health care resources does not outstrip the supply. In analogy with firefighting, flattening the curve accomplishes this by slowing the rate of burn as in a controlled burn. Delaying the peak works by temporarily extinguishing the flames, perhaps leaving the embers glowing. Both reduce the effective ***R***, sometimes through the same measures, and the fire will re-ignite in both when those measures are lifted. The difference lies in the shape of the epidemic curve afterward. Controlling, but not extinguishing, the burn reduces *S* and hence the reproductive number when the fire re-ignites. The overall infection attack rate AR—the fraction of the population that eventually becomes infected—is typically a sigmoidal function of ***R*** that is not very sensitive to ***R*** as long as it is above 1. For example, in the *S*–*I*–*R* model, $\varvec{R}_{0} = - ln\left( {1 - AR} \right)/AR$ (Ball 1983). Hence, flattening the curve does not significantly reduce the total number of people infected unless medical countermeasures can be developed during the delay it affords. In contrast, suppression works by driving infections to the (unstable) *I *= 0 fixed point. The reproductive number when control measures are lifted in this case will be the same as before they were imposed. Suppression may reduce morbidity and mortality substantially, but only if it is global and simultaneous or continues long enough for pharmaceutical interventions to be developed. When illness severity depends on demographics, mitigation measures are most efficient when they are demographically targeted; suppression measures are most efficient when they are spatially and temporally targeted.

Compartmental models can be elaborated (Hethcote 1994) by expanding the number of disease states (e.g., by adding compartments for exposed (*E*) or vaccinated (*V*)) or by partitioning the population by demographics such as age or location. Such models are well suited for representing demographically related heterogeneity in the course of illness or contact rates. The number of transition rates required to specify the model increases as the square of the number of partitions. At some point, depending on the modeler, it becomes natural to represent the model as a network of interacting partitions. Taken to the extreme, each host is in its own partition and the model is known as an individual-based model (Eubank 2004).

Individual-based models trade off the power to represent heterogeneity against computational complexity and the need for data to calibrate person–person contact rates as well as etiological parameters. Modelers have generally been slow to adopt individual-based models because of these challenges; rather, they felt that capturing individual heterogeneities was unnecessary. However, driven by important heterogeneities in the risk of HIV infection and the targeting of interventions, network models and individual-based models of small populations started to become popular around the turn of the century (Morris and Kretzschmar 1997; Black and Singer 1987). Individual-based models can represent not only biological, but also social heterogeneities, which are increasingly important aspects of pandemics. It is difficult to draw clear conclusions without such methods because individuals’ circumstances, perceptions, deliberations, and social conventions are rather centrally causal. Even a single individual’s behavior can carry out very important consequences. Hence, due to management of information and policies affecting individuals’ responses, biologically similar diseases and circumstances can have different outcomes that will not be captured by aggregate models.

The importance of developing useful models of infectious disease dynamics became abundantly clear in 2001 when five anthrax-laced letters were mailed to prominent senators and to media outlets, killing five people. Worries about bioterrorism led to increased federal spending and the expansion of federal agencies focused on protecting the public. The 9/11 attacks prompted then Vice President Cheney to propose vaccinating the entire population of the USA against smallpox. An NIH-sponsored forum held in 2001 concluded that models could be of great value in assessing the outcomes of Cheney’s and other strategies to reduce the threat of a smallpox attack (Kaplan et al. 2002; Halloran et al. 2002; Ferguson et al. 2003; Eubank 2004; Gani and Leach 2001).There was both optimism and skepticism about how, when, and whether modeling could be a useful tool for informing policy decisions.

As a result, the NIH National Institute for General Medical Sciences launched, in 2004, the Models of Infectious Disease Agent Study (MIDAS) to develop computational and mathematical models. An unusual clause in the MIDAS cooperative agreements stipulated that in the event of a national infectious disease emergency, the researchers would devote their attention to providing decision support to the Department of Health and Human Services. Then, as now, the emergence of a novel, highly lethal strain of influenza was perceived to be an imminent threat. MIDAS researchers prepared for their possible roles in an emergency by studying two questions of interest to pandemic preparedness planners:

1. In the event of an outbreak in, for example, Southeast Asia, should we commit our resources to containing the outbreak there, or should we reserve them to mitigate its eventual spread to the USA? (Ferguson et al. 2005; Longini et al. 2005).
2. Given limited supplies of antivirals, how effective would a strategy of targeted, layered containment (TLC) be in controlling an epidemic in the USA until a vaccine could be developed? (Halloran et al. 2008). Targeted, layered containment refers to implementation of several interventions that are individually ineffective, but potentially effective together.

Three modeling groups examined these questions and came to similar conclusions. The model that Imperial College developed was used as the basis for the recent analysis of the dynamics of SARS-CoV-2, the viral agent that causes COVID-19.

The results of these two studies were promising, but somewhat controversial at the time. The first study indicated that containment at the source was likely to succeed if outbreaks were detected early enough. Although the necessary global surveillance system did not exist, it seemed feasible. The TLC study indicated that a combination of thorough case detection and quarantine with isolation of contacts, careful targeting of antivirals, and aggressive social distancing measures adopted sufficiently early in an outbreak could, with high probability, slow the spread of disease enough so that health care resources would not be overwhelmed before sufficient quantities of pharmaceuticals could be manufactured and distributed.

Controversy centered on the feasibility of implementing aggressive social distancing measures, which included self-isolation, quarantine, and liberal leave policies at work. A 2004 Institute of Medicine panel considered the question, reaching equivocal conclusions, including that existing models were very promising but inadequate, at that time, to determine the impact of widespread social distancing measures (Mahmoud 2006; Hatchett et al. 2007; Markel et al. 2008).

What’s different now? Significantly, models have become more sophisticated, both computationally and in their relevance to policy decisions. The TLC models and results are certainly applicable to the current COVID-19 outbreak, although there are important differences:

- Not only is no vaccine available for SARS-CoV-2, but also no existing antivirals are known to be effective in treating illness or reducing transmissibility. The social distancing part of TLC is the only part relevant to the current COVID-19 epidemic.
- Unlike the influenza virus, SARS-CoV-2 is an emerging pathogen, not a novel variant of a well-known human pathogen. This has implications for public perceptions, development of diagnostic tests, and medical countermeasures. Moreover, the demographics of the illness, such as variations in the severity of illness, from asymptomatic to those requiring ventilator support, are almost unknown.
- The organization of society is rapidly changing due to near-universal access to high-bandwidth telecommunications and social media, affecting both the ability to communicate how to implement social distancing correctly and the dissemination of false information.

A beneficial result of these differences is that social distancing measures whose practicality was suspect then have been widely adopted today even in the absence of—or sometimes in opposition to—official guidance. School closings, community programs to support vulnerable people, state and county policies to require social distancing, as well as business and government support for telecommuting are widespread around the world.

Unfortunately, the lack of testing capacity and our poor understanding of variations in the severity of illness have made early case detection, isolation of infectious people, and quarantine of their contacts impossible. Thus, the brunt of suppressing COVID-19 falls on social distancing and the associated efforts to manage and control individual and population behaviors. There is no doubt that sufficient social distancing can suppress an outbreak of a droplet-borne respiratory disease. However, there is also no doubt that society could not continue to function if everyone withdrew to the home for the several weeks necessary for complete suppression. Indeed, the very notion of a society assumes that personal survival does not depend solely on individual decisions.

The Imperial College study addresses the question: If complete suppression is not feasible, what is the best strategy combining incomplete suppression and control that is feasible and leads to acceptable outcomes? The authors consider a strategy which cycles between maximum and minimum social distancing. Their most important assumptions are about the effectiveness of social distancing for reducing the reproductive number, the rate of case ascertainment, and the amount of infection before detection. The Imperial team, as usual, has taken great care to calibrate parameters of the disease model. To be sure, calibration has been hampered by the lack of testing, especially our poor understanding of the prevalence of asymptomatic infection, but the Imperial College team makes reasonable assumptions. However, the model’s reliance on a simplified picture of social interactions limits its extensibility to counterfactuals. The general nature of conclusions based on such a model can be expected to be similar to those of a simple compartmental model.

The Imperial team’s results are bleak, but they are consistent with other models that make similar assumptions (medRxiv 2020). The TLC study did not consider how and when to lift intervention measures, so the questions addressed by this study demonstrate important progress. They appear to have been influential in convincing policy-makers in the UK and the USA of the threat posed by COVID-19. They have stimulated discussion of novel mitigation strategies, although, once again, there is skepticism about the feasibility of on-again and off-again interventions. Such strategies not only depend on effective communication and adherence, but also on the premise of zero delay between sensing (e.g., ICU occupancy) and reacting (social distancing). The natural delay between imposing restrictions and seeing a drop in confirmed cases (as seen in Wuhan) will create social hysteresis loops. Also, it will be difficult for most officials to relax controls in the certain knowledge that many people will be infected.

Despite the progress, one must ask: Why we are still using models developed 15–20 years ago?

The timely nexus of the MIDAS program, commodity high-performance computing, the development of network science as a recognizable discipline, and the widespread adoption of distributed sensors like mobile GPS devices (a.k.a. smart phones) led to a burgeoning interest in epidemiological modeling. Individual-based models provide a high-resolution, mechanistic explanation of the reproductive number that can support principled modeling of the impacts of hypothetical social distancing strategies. However, marshalling the available evidence into a scalable, customizable model that is easy for non-computer specialists to use and that addresses a wide range of questions about a variety of strategies remains a challenge. Only a few of the existing efforts in this area have so far been able to bring the most powerful models to bear on COVID-19, but there are indications that more will be available in the coming days.

MIDAS is still in existence as of this writing, though the emergency clause has been dropped from its charter. The program has successfully nucleated a consortium of collaborating epidemiological modelers accustomed to playing an active role in decision support (Lofgren et al. 2014). CDC has now reached out to a stable of modelers to develop well-characterized models that inform CDC’s policy decisions. Contagions of all sorts—economic, social, and infectious disease—are among the most urgent issues of our time. The government should re-emphasize research into contagion in its sociotechnical context in a renewed, invigorated, and broadened MIDAS-style thrust for the future.
